# PET Molecular Imaging in Breast Cancer: Current Applications and Future Perspectives

**DOI:** 10.3390/jcm13123459

**Published:** 2024-06-13

**Authors:** Sanaz Katal, Michael J. McKay, Kim Taubman

**Affiliations:** 1 Medical Imaging Department, St. Vincent’s Hospital Melbourne, Fitzroy, VIC 3065, Australia; sanaz.katal@svha.org.au; 2 Northwest Regional Hospital, University of Tasmania, Burnie, TAS 7320, Australia; michael.mckay@ths.tas.gov.au; 3 Northern Cancer Service, Northwest Regional Hospital, Burnie, TAS 7320, Australia

**Keywords:** breast cancer, PET, PET/CT, PET/MRI, estrogen receptor (ER), progesterone receptor (PR), androgen receptor (AR), human epidermal growth factor receptor 2 (HER2), FDG, FAPI, FES, FDHT, artificial intelligence (AI), machine learning (ML), deep learning (DL)

## Abstract

Positron emission tomography (PET) plays a crucial role in breast cancer management. This review addresses the role of PET imaging in breast cancer care. We focus primarily on the utility of 18F-fluorodeoxyglucose (FDG) PET in staging, recurrence detection, and treatment response evaluation. Furthermore, we delve into the growing interest in precision therapy and the development of novel radiopharmaceuticals targeting tumor biology. This includes discussing the potential of PET/MRI and artificial intelligence in breast cancer imaging, offering insights into improved diagnostic accuracy and personalized treatment approaches.

## 1. Introduction

Breast cancer (BC) is the most frequent cancer diagnosed in women globally, comprising 11.7% of total cancer cases. The highest incidence rates (>80 per 100,000) are observed in Australia/New Zealand, Western Europe, North America, and North Europe [1]. While the incidence rate of BC has increased by 0.5% yearly between 2010 and 2019, the mortality rate has declined at the average rate of 1.3% yearly from 2011 to 2020. This decline is attributed to screening and therapy advancements [2]. Nonetheless, despite the improved survival rate, BC remains the fifth leading cause of cancer-related death in Australia [3].

Accurate initial staging, restaging, and evaluation of therapy response are essential for planning optimal breast cancer management. Positron emission tomography (PET) has proven to be an invaluable molecular imaging technique in the management of patients with BC. In this review, we will discuss the current and potential applications of PET/CT in breast cancer using different radiotracers, such as 18F-fluorodeoxyglucose (FDG), 16α-18Ffluoro-17β-oestradiol (FES), and novel radiopharmaceuticals, PET/MR, and the application of artificial intelligence (AI) and deep learning (DL) methods in BC.

### 1.1. FDG PET/CT Applications

#### 1.1.1. Initial Diagnosis

Early detection is crucial for improving the prognosis and survival of BC patients. Imaging plays a pivotal role in screening and primary detection, utilizing techniques such as mammography, ultrasound (US), and magnetic resonance imaging (MRI). Currently, mammography remains the standard tool for the detection of primary breast tumors. Either mammography or ultrasound can be applied to assess alterations in the morphology of breast tissue [4].

**Figure 1:**
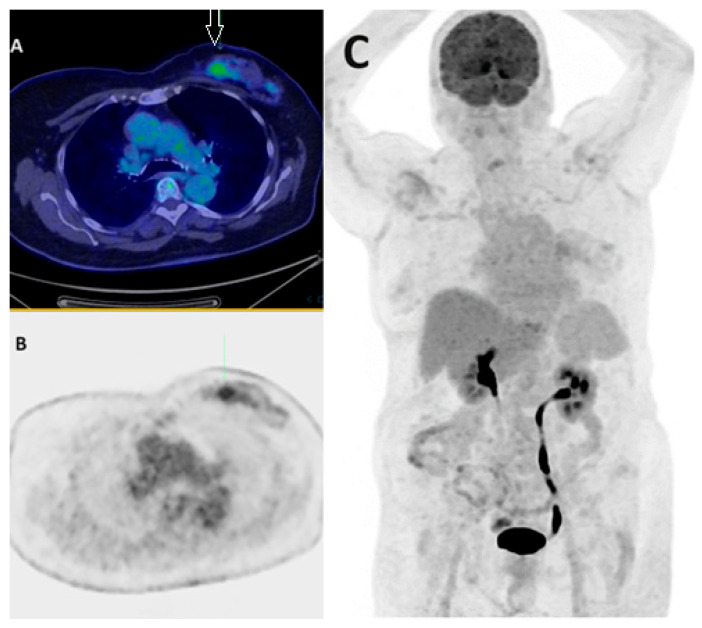
Lower FDG avidity in certain BC subtypes. Fused axial PET/CT (**A**), axial PET only (**B**), and MIP PET/CT (**C**) demonstrate low-grade FDG uptake in biopsy-proven left breast lobular carcinoma (arrow) in a 55-year-old patient. The lower SUVmax of invasive lobular carcinoma (ILC) primary tumors compared to invasive ductal carcinomas (IDCs) is attributed to factors such as lower tumoral cell density and reduced proliferation rates.

According to the current literature and guidelines, PET/CT cannot be routinely recommended as a primary diagnostic tool for breast cancer due to its high cost and limited sensitivity [5,6]. PET has a low sensitivity for diagnosis of early-stage BC, especially in lesions less than 5–10 mm, attributed to partial volume effects and limited spatial resolution. In addition to small tumor size, certain biological characteristics of BC, such as proliferation rate, tumor grade, histological subtype, and hormone receptor status, may affect its detection ability. Lobular carcinoma, tumors with low proliferation as indicated by the Ki67 index, and grade one or two tumors tend to display lower FDG avidity, potentially leading to false negatives on FDG PET [7]. Additionally, FDG uptake is negatively correlated with hormonal receptor status, with well-differentiated estrogen/progesterone receptor-positive (ER+, PR+) tumors demonstrating lower FDG avidity than ER-/PR- tumors [8]. In a cohort of 74 patients with grade 1–2 ER+ breast cancer, FDG PET findings resulted in inadequate staging in 22.9% of cases, highlighting the limitations of PET/CT in certain subtypes and scenarios [9] (Figure 1).

**Figure 2:**
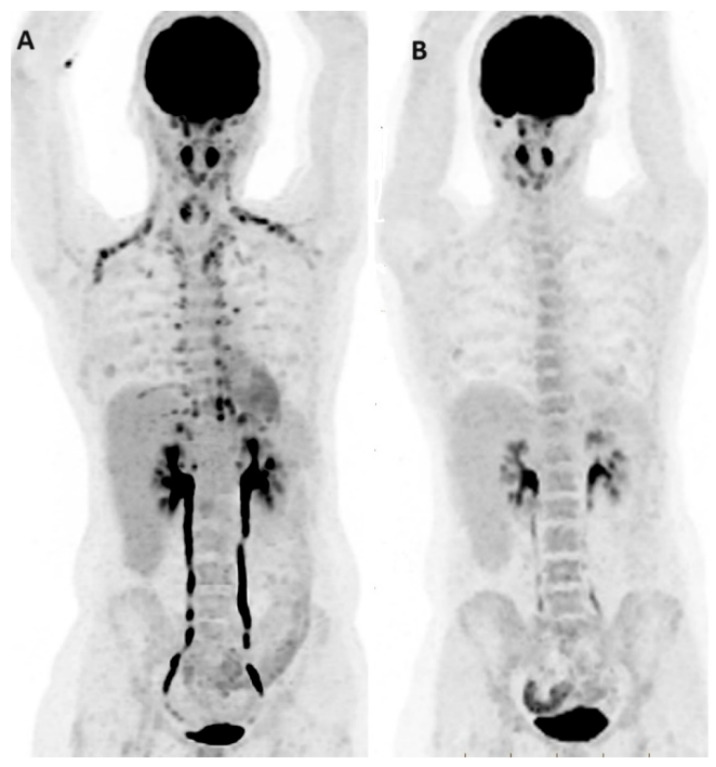
Physiologic brown fat uptake. MIP FDG PET/CT imaging (**A**) demonstrates extensive brown fat uptake, leading to a technically limited, non-diagnostic study in a 45-year-old patient. The patient returned for a repeat PET scan (**B**) on another date post-administration of beta-blockers.

Apart from low sensitivity, the specificity is also somewhat limited. FDG lacks specificity for malignancy, as it can show increased uptake in inflammatory processes and benign tumors such as fibroadenomas. Furthermore, various physiological processes may limit cancer detection, including brown adipose tissue, cerebral glucose uptake, recent ipsilateral vaccinations, and muscle activity (Figure 2).

Nonetheless, despite these limitations, PET/CT has a sensitivity and specificity in BC, ranging from 48 to 96 and 73 to 100%, respectively [10]. More importantly, functional imaging with PET offers additional insights into tumor histopathologic features, grade, and hormonal receptor expression status, contributing to prognostication and management decisions. Key FDG PET parameters, such as the maximum standardized uptake value (SUVmax) of the primary tumor, provide valuable predictive and prognostic information, enhancing personalized medicine approaches. The SUVmax of the primary tumor is significantly higher in ER-negative, PR-negative, HER2-positive, and Ki-67-positive BC. This pattern is consistent across clinical subtypes, with luminal subtypes exhibiting lower FDG uptake and triple-negative breast cancers (TNBCs) showing higher uptake [11]. Tumor heterogeneity, measured using texture analysis in FDG PET/CT, serves as another valuable tool directly correlated with tumor aggressiveness [12]. These advancements highlight the potential of PET/CT, combined with innovative approaches, to offer comprehensive insights for more effective breast cancer management. Moreover, the integration of AI into FDG PET imaging shows promise as an adjunct to further improve diagnostic, predictive, and prognostic accuracy [13].

Dedicated breast imaging, such as positron emission mammography (PEM), which is far more sensitive than the whole-body FDG PET/CT for the detection of primary tumors, could also be considered when MRI is contraindicated [14].

Overall, FDG PET is not deemed suitable either for the screening or detection of primary tumors or screening. However, it can be regarded as a valuable supplement to conventional imaging procedures [5].

#### 1.1.2. Initial Staging

Accurate initial staging is crucial for optimizing therapeutic planning and clinical outcomes in newly diagnosed BC. When compared to conventional imaging techniques, FDG PET offers superior performance in detecting extra-axillary nodal and distant metastases. Although current oncologic practice guidelines do not recommend 18F-FDG PET/CT for the initial staging of early BC [15,16], as of the 2022 National Comprehensive Cancer Network (NCCN) guidelines, PET/CT is suggested for systemic staging in newly diagnosed stage III or when standard staging studies provide non-diagnostic or suspicious results [17]. The guidelines emphasize that FDG PET/CT is not indicated for patients with clinical stages between I and operable III unless suspicion of metastatic disease exists. This is attributed to the high false-negative rate for small (<1 cm) and/or low-grade disease, the high false-positive rate in those without locally advanced disease, the low sensitivity for detecting axillary nodal metastases, and the low probability of detectable metastatic disease in these patients [18].

**Figure 3:**
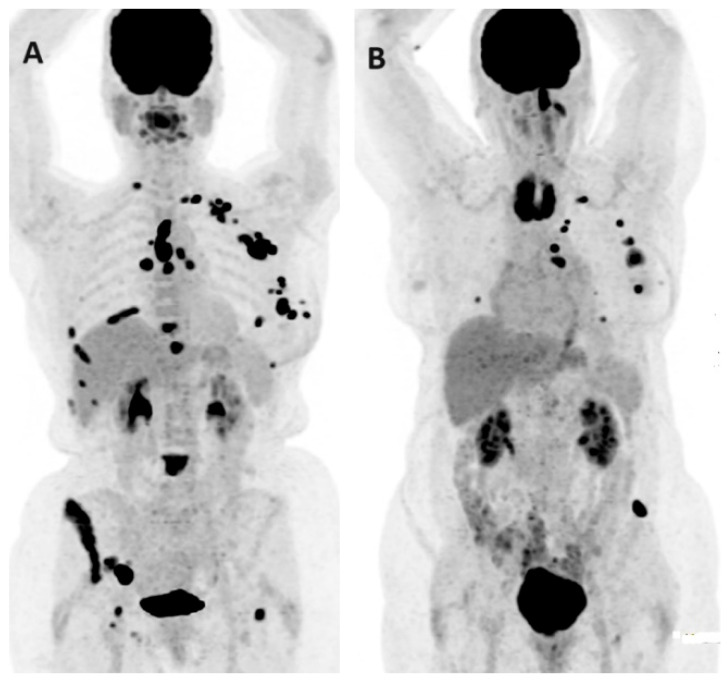
FDG PET/CT in BC initial staging. PET/CT MIP images of two breast cancer patients (**A**,**B**) demonstrate intensely FDG-avid multifocal left breast cancer, with an unexpected extensive burden of regional and non-regional nodal disease as well as multifocal distant osseous and pulmonary metastases.

Despite the recommendations, however, the precise clinical stage at which PET/CT can be performed with a cost-effective balance remains unclear [19], with emerging evidence over recent decades suggesting a potential role for FDG PET/CT in the initial staging and clinical management of all BC patients. The literature indicates a significant yield not only for high-risk patients (locally advanced or inflammatory BC) but also for intermediate-risk patients with clinical stage IIB disease or higher. Some reports even demonstrate a non-negligible proportion of upstaging by FDG PET/CT in stage I (11%) and stage II (20%) disease [19]. In a meta-analysis with 4276 patients, FDG PET, PET/CT, or PET/MRI for initial staging of BC resulted in changes in staging and management in 25% and 18% of patients, respectively [19]. Furthermore, the additional information provided by PET/CT about tumor type and histology may justify initial staging with PET/CT at any stage, potentially leading to substantial changes in the management of BC patients [20]. Thus, PET imaging may deserve to be added to the evaluation of newly diagnosed patients in some instances (Figure 3).

##### Axillary Lymph Node Staging

Axillary lymph node status is one of the most important prognostic factors in BC. Sentinel lymph node biopsy (SLNB) remains the gold standard of lymph node evaluation in BC, where a positive SLNB prompts axillary lymph node (ALN) dissection or, more frequently, axillary radiation therapy. Recent meta-analyses have reported that the performance of PET/CT is inferior to SLNB for nodal staging in BC. However, the high specificity of 94% of PET/CT in axillary nodal assessment indicates its potential usefulness in specific clinical scenarios [21]. Detecting a positive lymph node on PET/CT in a patient with suspected advanced disease might justify proceeding directly with ALN dissection, thereby potentially avoiding the need for SLNB. Nonetheless, PET/CT is not recommended to replace SLNB for nodal staging in BC, owing to its lower sensitivity for micrometastases, lower avidity of some primaries, and challenges in larger patients [22]. Therefore, SLNB remains the method of choice for diagnosing ALN involvement.

**Figure 4:**
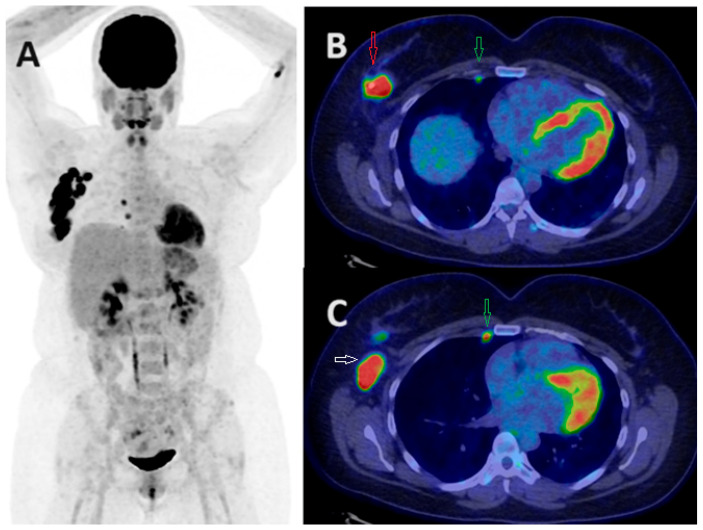
FDG PET/CT in BC nodal staging. The PET/CT MIP (**A**) and axial fused PET/CT (**B**,**C**) images demonstrate a hypermetabolic right breast primary (red arrow) with multiple hypermetabolic ipsilateral right level I/II axillary (white arrow) and internal mammary (green arrow) nodal metastases.

**Figure 5:**
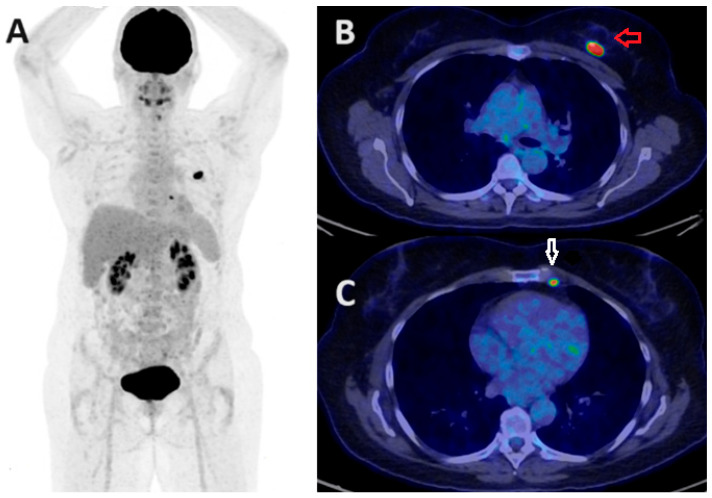
FDG PET/CT in BC nodal staging. The PET/CT MIP (**A**) and axial fused PET/CT (**B**,**C**) images demonstrate high FDG uptake within the left breast primary (red arrow) with solitary ipsilateral internal mammary nodal disease (white arrow) in a 55-year-old patient referred for staging of a newly diagnosed TNBC. Internal mammary node positivity is more common in centrally or medially located BCs.

The assessment of internal mammary (IM) node metastasis has also undergone a thorough examination with PET/CT. In a retrospective analysis of 249 patients, PET/CT had a high positive predictive value (PPV) of 87.1% in diagnosing IM metastasis among stage III BC [23]. Another study suggested that PET/CT had a higher area under the curve (AUC) of 0.87 compared to US, with an AUC of 0.83 for detecting IM node metastasis [24]. Although only 10% of BC patients had positive IM nodes on FDG PET/CT, a positive IM node on PET indicated a very high likelihood of malignant involvement on US-guided FNA [25]. In a very recent prospective study by Garcia et al., the detection rate of IM chain infiltration was 34% in stage ≥IIB BC, which allowed tailored therapy in many cases [26]. Hence, FDG PET could be applied as a non-invasive tool for the evaluation of IM nodes. More studies are still needed in this regard (Figure 4 and Figure 5).

##### Distant Metastasis

Up to 30% of BC patients may experience distant relapse after initial treatment, and this depends on factors such as the stage, subtype, grade, Ki67, receptor negativity, and the presence of lymph node metastasis [27,28]. Conventional imaging modalities, including CT, MRI, and bone scans, are widely used to diagnose distant metastases in BC patients.

FDG PET/CT emerges as a potentially highly accurate tool in diagnosing distant metastasis in locally advanced BC and stage IVA, IB, IVC, and IVD. Its capability to detect unsuspected distant metastases, including distant nodes, pleura, liver, spleen, adrenal, and pelvis [29], suggests that incorporating FDG PET/CT into staging protocols may result in upstaging many patients with high-risk BC, prompting adjustments in their treatment plans [20]. It is believed that patients with stage III and IV BC would benefit the most from FDG PET/CT imaging for initial staging, as they have the highest likelihood of distant metastatic disease at the time of diagnosis [19].

Current guidelines recommend PET/CT as a valuable alternative in the evaluation of distant metastasis in BC, particularly when conventional modalities yield inconclusive results or benign lesions or are not applicable. In a meta-analysis with nine studies, whole-body MRI and FDG PET/CT demonstrated nearly equivalent sensitivity (86% vs. 85%) and specificity (97% vs. 96%) for distant staging in cancer patients [30]. Other studies have shown that PET/CT outperforms conventional imaging methods, exhibiting sensitivity and specificity ranging from 0.96 to 0.99 and 0.95, respectively [31]. This body of evidence highlights the established and evolving role of PET/CT as a promising tool for detecting distant metastasis in BC.

By detecting distant metastases, PET/CT imaging has the potential to impact the treatment plan, leading to a shift from neoadjuvant or surgical therapy to palliative therapy in stage IIB and III breast cancer, particularly in younger patients [32]. In a study involving 134 patients with stage I to IIIC, PET/CT identified distant metastasis in 17% of asymptomatic stage BC patients who were under 40 years old [33]. In another study by Yarabas et al. [34], the overall upstaging rates were 18.6% for patients with initial stage IIA, 30% for those with stage IIB, and 46.3% for those with stage IIIA BC after PET/CT. This underscores the valuable role of PET/CT in guiding treatment decisions and altering therapeutic strategies based on more accurate staging information. Figure 3 depicts two cases of BC that were upstaged following PET/CT.

Bone represents the most common site of BC metastasis, occurring in approximately 65% of metastatic cases [35]. Due to its acceptable sensitivity and cost-effectiveness, bone scintigraphy remains the standard procedure for detecting bone metastasis in BC patients [36]. PET/CT is increasingly considered a valuable alternative for detecting distant metastasis in BC, particularly bone involvement. Numerous studies have shown the superiority of PET/CT over bone scintigraphy in detecting bone metastases, demonstrating enhanced sensitivity and specificity, especially for lytic or mixed bone metastases and bone marrow involvement. In a meta-analysis by Rong et al. [37], with seven studies and 668 patients, PET/CT exhibited higher sensitivity and specificity compared to bone scintigraphy (93% and 99%, respectively).

It is reported that FDG PET/CT may be less sensitive for purely sclerotic bone metastases, which are characteristic of BC. Therefore, some clinicians may choose to include bone scintigraphy even in patients who have undergone FDG imaging. However, it is not uncommon to observe FDG-avid sclerotic metastases. In addition, a careful assessment of the CT component often allows these osteosclerotic lesions to be detected on FDG PET/CT. Moreover, many of these studies were retrospective, where many patients were not treatment naïve. In this setting, FDG-negative osteosclerotic lesions do not necessarily indicate false negativity of FDG PET but may represent bone healing in responders [38].

In lung parenchyma, PET has limited sensitivity for subcentimetric pulmonary nodules due to partial volume effects and respiratory motion artifacts. Consequently, a comprehensive assessment of the CT component is imperative to detect small, non-FDG-avid pulmonary nodules. However, despite its clinical utility in various contexts, the non-diagnostic low-dose CT component of a hybrid PET/CT is less effective in detecting these nodules compared to a dedicated chest CT [38].

In summary, the choice of imaging for staging BC should be tailored to the individual characteristics of the tumor and patient, considering factors such as molecular subtype, Ki67 index, grade, receptor status, and clinical stage. The tumor grade is a particularly important factor to consider when deciding on the imaging modality for BC staging. For instance, FDG PET/CT is not routinely recommended for staging low-grade BC, especially for those with stage I and IIA disease, given the low rates of upstaging in these patients. In a cohort study with 74 patients, FDG PET/CT failed to correctly stage 16/70 (22.9%) of grade 1–2 ER-positive BC patients [9]. Additionally, a meticulous evaluation of the low-dose CT component and the utilization of complementary imaging tools, such as bone scans or brain MRI, may be important, especially when FDG PET/CT yields negative results in high-risk patients.

#### 1.1.3. Evaluation of Response

Neoadjuvant chemotherapy (NAC) is a standard therapy for BC patients with locally advanced and inflammatory BC. Recently, the role of NAC has expanded from its initial use in inflammatory BC to now encompass early-stage BC (especially HER2+), aiming to induce tumor shrinkage and facilitate less extensive surgery. Previous studies have shown superior disease-free survival and overall survival in BC patients who achieve a pCR (pathological complete response) post-NAC [39]. Accordingly, predicting the response to NAC and distinguishing responders from non-responders early in the treatment course has become crucial for adjusting treatment strategies. In order to avoid deficient or excessive chemotherapy and inappropriate surgical decision making for BC patients before surgery, an effective method to evaluate response is appealing.

While conventional methods such as ultrasound, mammography, and MRI are commonly used to assess NAC response, encouraging findings support the accuracy of PET/CT in this context [40]. A higher baseline glycolytic activity on FDG PET/CT and a larger subsequent decrease in SUVmax following early NAC cycles are predictors for favorable histopathological outcomes after the completion of NAC [41]. Notably, early metabolic non-response after NAC has been associated with pathological non-responders and poorer prognosis, particularly in ER-positive/HER2-negative cases [42]. A meta-analysis by Maganga et al. with 745 patients has shown that FDG PET is valuable for early monitoring of BC responders and non-responders after 1 or 2 NAC cycles, with moderate sensitivity (80.5%) and specificity (78.8%) [43]. The absence of FDG uptake post-therapy (in the absence of masses on corresponding CT) is indicative of improved survival rates in patients with locally advanced BC [19]. Multiple studies have shown the utility of FDG PET imaging for early response assessment in specific subtypes of BC, including ER-positive, HER2-positive, and triple-negative BCs [38,44]. However, tumors with low FDG avidity, such as lobular carcinoma, may not benefit from FDG PET for response monitoring.

A meta-analysis by Tian et al. recommends a combination of imaging modalities to precisely predict pCR [45]. The analysis of 22 studies with 1119 patients with known BC demonstrated pooled sensitivity and specificity of FDG PET/CT at 81.9% and 79.3%, respectively. The diagnostic odds ratio was calculated at 17.35, confirming the utility of FDG PET/CT in predicting treatment response. However, variations in sensitivity and specificity across studies point to the importance of a tailored approach based on individual patient characteristics and tumor biology [46].

**Figure 6:**
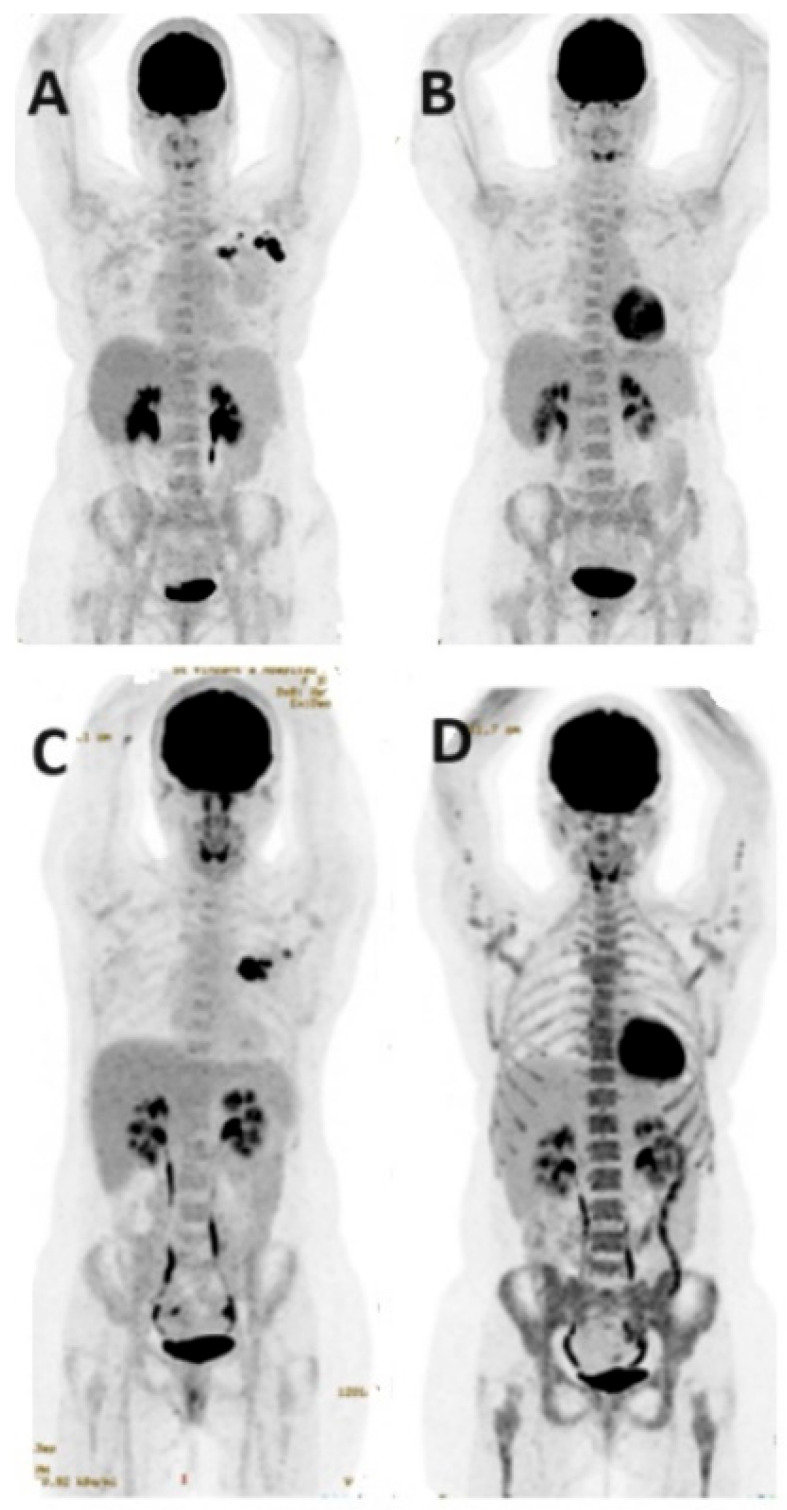
FDG PET/CT in response assessment. Baseline PET/CT MIP images (**A**,**C**) show FDG-avid left BC and ipsilateral axillary nodal metastases in two patients before neoadjuvant chemotherapy (NAC). Post-NAC PET/CT MIP images (**B**,**D**) demonstrate an excellent treatment response, with significantly reduced FDG uptake in the primary and axillary nodal metastases following neoadjuvant chemotherapy.

Response assessment in metastatic BC presents additional challenges. While FDG PET/CT has demonstrated superiority in early response evaluation for metastatic BC, there is a lack of prospective studies investigating its clinical efficacy in this group [47,48]. The correlation between enhanced diagnostic accuracy in response monitoring using FDG PET and improved survival outcomes in these patients remains uncertain. Theoretically, the increased sensitivity and accuracy of PET in detecting disease progression or regression holds promise for guiding therapeutic options, potentially improving survival rates among metastatic BC patients. However, achieving international consensus on response assessment using PET necessitates further exploration through large prospective clinical trials in this field (Figure 6).

#### 1.1.4. Recurrence

The majority of early BC patients undergo local excision surgery and postoperative radiotherapy, with a 5-year relative survival rate of approximately 98% for those diagnosed with localized disease. While locoregional recurrence carries a poor prognosis, early detection and staging of recurrence can improve survival [49].

FDG PET is a well-established tool in recurrent BC, with at least equal accuracy as MRI [50]. Numerous studies have shown that FDG PET compares favorably with other imaging tools, such as bone scan, CT, or MRI [51,52,53,54]. Two meta-analyses and systematic reviews on BC recurrence detection using PET were published in 2010 [55,56]. The first analysis favored MRI and PET (and/or PET/CT) over ultrasound and CT, without significant differences between MRI and PET. The second analysis showed higher sensitivity in hybrid PET/CT compared to CT, and PET/CT outperformed PET alone in sensitivity. No significant differences were found between PET/CT and MRI, although only one whole-body MRI study was included. In another meta-analysis published in 2016 by Xiao et al. [57] involving 26 studies and 1752 patients, PET/CT demonstrated pooled sensitivity of 90% and specificity of 81% for detecting recurrent BC.

Previous studies have shown that PET/CT has high diagnostic performance in identifying recurrence among asymptomatic BC patients with rising tumor markers (CA 15–3 and CEA). In a study by Grassetto et al. [58], PET/CT detected recurrence in 45% of BC patients with elevated markers and negative conventional imaging results. In a meta-analysis in 2012, PET had a sensitivity of 87.8%, specificity of 69.3%, and diagnostic accuracy of 82.8% in detecting recurrence in BC with elevated markers [59]. Of note, PET/CT can identify recurrence in clinically suspicious cases, even when tumor markers are negative.

PET/CT also plays a crucial role in patients with confirmed or suspected recurrence. The advantage of PET/CT is the ability to screen the entire body in a single session with high sensitivity and reasonable specificity. It can determine whether the recurrence is localized or not, which potentially impacts patient management. In a study with 56 recurrent BC patients considered for curative surgery, PET/CT uncovered previously undetected distant lesions in 25 cases (45%) [60]. It changed the management in 27 patients (48%) by detecting more extensive locoregional disease or distant metastases.

Current guidelines recommend against routine FDG PET/CT imaging for surveillance of BC in asymptomatic patients due to cost and insufficient evidence; however, this may change. A recent retrospective study published in 2023 with 1681 patients suggests that surveillance PET/CT has an excellent diagnostic performance in detecting clinically unexpected recurrent BC after curative resection [61]. However, further studies, including randomized clinical trials, are needed to assess the cost-effectiveness and survival benefits of routine surveillance PET/CT. Moreover, while PET/CT is more accurate than conventional imaging in identifying recurrence, its accuracy may be limited in certain cases, such as lung nodules, liver disease, small recurrent tumors, and small metastases, particularly in the brain [62].

In conclusion, PET/CT is not currently recommended for the routine follow-up of BC patients but remains the most valuable tool for detecting recurrence or metastasis in patients with confirmed or suspected recurrence. Suspected cases of recurrence with rising markers or equivocal/suspicious clinical or radiologic recurrence are considered the best candidates for PET/CT. FDG PET/CT is also a potential tool for defining the total disease burden in cases of locoregional recurrence and distant metastasis.

### 1.2. Beyond FDG

As personalized medicine gains traction, particularly with the advancement of molecular-targeted therapy and theranostics, the role of molecular imaging has grown substantially across various cancers, including BC. Molecular imaging with PET/CT has become essential for identifying potential targets within the tumor microenvironment. It aids in the selection of patients who are most likely to benefit from novel molecular-targeted therapies, optimizing therapeutic efficacy and reducing adverse effects. This paradigm shift underscores the evolving and integral role of PET/CT in the era of personalized and targeted BC management.

While FDG remains an invaluable tool in BC imaging, several novel PET radiotracers show substantial potential to revolutionize clinical breast oncology. This potential arises from addressing FDG’s limitations, including lower sensitivity for detecting small, low-grade tumors, specific subtypes of BC, and axillary nodal metastasis, as well as its propensity for false-positive results in benign and inflammatory lesions [17]. Recent studies have investigated various novel PET radiopharmaceuticals, emphasizing their ability to in vivo evaluate estrogen receptor (ER), fibroblast activation protein (FAP), gastrin-releasing peptide receptor (GRPR), human epidermal growth factor receptor-2 (HER2), cell proliferation, chemokine receptor, and amino acid metabolism.

Although not currently approved for clinical use in BC patients, these innovative imaging agents hold promise for roles in diagnosis, staging, management, and targeted therapies. In this context, we will review some of these tracers designed for different molecular targets, including 68Ga-fibroblast activation protein inhibitor (FAPI) for FAP, 89Zr-Df-trastuzumab for HER2, and 18F-fluoroestradiol (FES) for ER.

#### 1.2.1. Fibroblast Activation Protein Inhibitor—FAPI

FAP, a type II transmembrane glycoprotein, plays a crucial role in tumor biology by influencing various hormones and extracellular matrix components. Overexpression of fibroblast activation protein (FAP) in cancer-associated fibroblasts (CAFs) is prevalent in human epithelial cancers with high-grade desmoplasia, such as breast, colon, and pancreatic carcinomas. Higher FAP expression serves as a marker for aggressive tumor behavior and is associated with a poor prognosis. Due to its low expression in most normal organs, FAP becomes an attractive target for both imaging and endo-radiotherapy. This characteristic has driven the development of several FAP-targeting radiopharmaceuticals.

In recent years, molecular PET/CT imaging with radiolabeled FAP inhibitors (FAPIs) has gained an important role in several cancers. To exploit FAPIs as a diagnostic and potential theranostic agent, different types of FAPI-based radiopharmaceuticals have been investigated, either labeled with [68Ga] Ga, [177Lu] Lu, or [225Ac] Ac. The most promising candidate, 68Ga-FAPI, exhibited the most favorable characteristics with an ultrahigh affinity for FAP-expressing cells. 68Ga-FAPI PET/CT provides remarkably high uptake and image contrast in various tumors, with the highest uptake (average SUVmax > 12) in lung, breast, and esophageal cancer, cholangiocarcinoma, and sarcoma. Interestingly, it could also identify liver metastases as small as 1 cm in diameter [63].

Numerous studies are underway to explore the evolving role of FAPI PET in assessing BC, hinting at its potential superiority over FDG PET. Consistent findings indicate that 68Ga-FAPI PET/CT offers remarkably high tumor uptake alongside minimal uptake in normal organs, thereby yielding high-contrast images [64]. More importantly, the application of FAP-targeted theranostics is attractive, supported by studies using [90Y]-FAPI SPECT to confirm tracer accumulation in metastatic sites, resulting in a significant reduction in pain medication without side effects [65]. Further research is warranted to delve deeper into this promising approach, potentially offering a new avenue for patients who have progressed despite conventional treatments.

Several studies have reported that 68Ga-FAPI PET/CT outperformed FDG PET/CT in detecting both primary and metastatic breast cancer, attributed to lower background activity and higher uptake in subcentimetric lesions [66]. The preliminary experience with using FAPIs in breast cancer has been promising for several reasons [67]. Firstly, FAPIs revealed more lesions with much higher tumor-to-background ratios compared with FDG. Secondly, FAPI uptake was not correlated with histopathological features and molecular subtypes, being equally increased in all types of breast cancer. This is particularly important in BC cases where there is low FDG uptake, such as lobular cancer, or in those with HER2-positive, luminal A, and luminal B disease. Lastly, with substantial theranostic potential, 68Ga-FAPI PET/CT could become pivotal in predicting the pathological response of breast cancer patients, a promising area for further research.

#### 1.2.2. Hormone Receptor Imaging

Over 70% of BC patients present with hormone-receptor-positive disease, characterized by the expression of estrogen receptor (ER+), progesterone receptor (PR+), or both, and will typically undergo endocrine therapy [68]. The ER status of BC patients is an important prognostic indicator for disease-free survival and overall mortality due to its significant impact on treatment decision making. Therefore, establishing the hormonal status of tumors is paramount.

While tumor biopsy and immunohistochemistry serve as the gold standard for determining receptor expression, their invasive nature, potential sampling errors, variations in receptor expression, and discordant ER expression between primary and metastatic lesions present challenges in treatment planning. To address these issues, non-invasive imaging methods have been developed.

The use of PET/CT with radiotracers like 16α-[18F] fluoro-17β-oestradiol ([18F] FES) and 16β-[18F] fluoro-5α-dihydrotestosterone ([18F] FDHT) has emerged as a valuable approach. These radiotracers enable the non-invasive visualization of ER and androgen receptor (AR) status in tumor lesions within breast cancer patients, offering insights that can aid in treatment planning and monitoring.

##### Estrogen Receptor Imaging—FES

Non-invasive imaging using 18F-fluoroestradiol (FES) PET/CT proves invaluable in assessing estrogen distribution and binding across multiple sites, offering simultaneous confirmation of metastases. The unique capability of FES PET/CT positions it as a powerful tool for predicting treatment response in BC patients. Providing comprehensive whole-body information on the heterogeneity of ER expression in BC metastases, FES PET/CT enhances the understanding of BC dynamics and supports the development of personalized treatment strategies.

FES received FDA approval in May 2020 for the detection of ER-positive lesions as an adjuvant to biopsy in recurrent and metastatic BC. Prior studies have demonstrated the role of FES PET in assessing in vivo ER expression, predicting response to hormone therapy and adjuvant chemotherapy, evaluating the efficacy of ER blockade, and aiding in personalized treatment strategies.

Functioning as a diagnostic tool, 18F-FES PET significantly improves diagnostic clarity in 88% of cases and prompts therapeutic adjustments in 48% of patients facing clinical dilemmas, such as equivocal or conflicting results in conventional workups.

In terms of predicting treatment response, 18F-FES PET proves effective in monitoring PR expression during treatments with ER down regulators like fulvestrant, offering the potential for prognostic assessment and therapy decisions. A lack of substantial reduction in 18F-FES PET activity (<75%) post-fulvestrant treatment is associated with early disease progression.

However, there is still limited information available regarding the diagnostic utility of this receptor-specific tracer. While some studies indicate its low sensitivity in evaluating BC liver metastases, others highlight its significant diagnostic impact in identifying BC bone metastases. Overall, it remains unclear whether 18F-FES PET/CT could be recommended as a primary imaging module in ER+ BC patients with suspected distant metastases and whether it could be favored over 18F-FDG PET/CT, particularly in specific scenarios.

##### Progesterone Receptor Imaging

18F-fluorofuranyl norprogesterone (18F-FFNP), a progesterone analog relying on the presence of progesterone receptors (PRs), is another novel PET tracer that is under investigation. This radiotracer can assess the PR expression of individual BC lesions. Therefore, considering 18F-FFNP PET as a non-invasive test to predict patient response to endocrine therapies and monitor PR levels following hormone treatment appears reasonable [69]. Studies using 18F-FFNP PET scans in ER-positive BC before and immediately after a one-day oestradiol challenge have suggested that the change in 18F-FFNP uptake in a tumor after an oestradiol challenge serves as a highly predictive indicator of responses to endocrine therapy in ER-positive BC [70,71]. Consequently, 18F-FFNP holds promise as a valuable biological agent for predicting and monitoring treatment efficacy in PR-positive BC. However, it is premature to reach a definite conclusion at this stage.

#### 1.2.3. Human Epidermal Growth Factor Receptor 2 (HER2)

Human epidermal growth factor receptor 2 (HER2), belonging to the tyrosine kinase receptors family, plays a crucial role in cell growth, survival, and development of metastasis [72], with overexpression occurring in approximately 20–30% of all BC patients. Historically, HER2-positive BC had the worst prognosis among BC subtypes until the introduction of trastuzumab, an FDA-approved monoclonal antibody targeting HER2/neu cancer cells [73,74]. This breakthrough has significantly improved patient survival. However, most BC patients eventually develop resistance during the treatment course [75]. The dynamic nature of HER2 status, varying across different tumor sites, further complicates matters. While repeated biopsies are encouraged to monitor HER2 status changes, this is not always practical. The HER2 expression in BC can be measured using PET imaging with radiolabeled monoclonal antibodies (mAbs) like [89Zr] trastuzumab. This non-invasive approach allows for the detection and quantification of HER2 expression throughout the entire body, providing valuable insights into the effectiveness of targeted immunotherapies in BC patients [76]. Trastuzumab and related mAbs have also been radiolabeled with various other diagnostic and therapeutic radionuclides, including 64Cu, 68Ga, and 177Lu. Several studies have suggested that PET/CT imaging using these novel tracers serves as a non-invasive method for whole-body assessment of HER2 status, potentially guiding treatment decisions in HER2-positive BC patients. Despite these promising results, further clinical trials are necessary to fully assess the significance of HER2-targeted PET/CT in BC management.

#### 1.2.4. Other Tracers

##### Prostate-Specific Membrane Antigen (PSMA)

Prostate-specific membrane antigen (PSMA) is an integral membrane protein that is commonly overexpressed in prostate carcinomas and the tumor-associated neovasculature of various solid tumors, including breast carcinoma [77,78]. This overexpression makes PSMA a potential target for therapies, which could be considered as an option for BC patients refractory to standard treatments. The exploration of therapies directed at PSMA expression opens new avenues for personalized and targeted approaches in the management of BC.

##### Chemokine Receptor 4 (CXCR4)

Chemokine Receptor 4 (CXCR4) is a chemokine receptor that is often overexpressed in invasive BC, contributing to tumor aggressiveness [79]. The use of CXCR4-directed PET, specifically with 68Ga-Pentixafor PET/CT, in patients with primary and recurrent BC may play a role in prognostication and help identify potential candidates for therapies targeting CXCR4. It is noteworthy, however, that the detectability of tumors using this method is significantly lower compared to FDG PET. Therefore, while it may have specific applications in assessing CXCR4-related aspects in BC, it may not be suitable as a general diagnostic tool for imaging BC.

##### Somatostatin Receptors (SSTRs)

All five subtypes of somatostatin receptors (SSTRs) are variably expressed in primary BC, with a positive correlation observed between several receptor subtypes and hormone receptor-positive tumors [80]. This suggests that SSTR imaging could potentially play a role in patients with poor FDG uptake, providing valuable guidance for hormonal therapy. However, a thorough study comparing the effectiveness of 68Ga-DOTATATE with 18F-FES for this specific indication is needed [81]. While octreotide (OTA) has shown superiority to FDG in staging ER+/PR+ BC, DOTATATE may have a distinct role in assessing HR status and guiding treatment decisions; further evaluation of this is warranted.

##### Androgen Receptor

Androgen receptor (AR) is highly expressed in BC, particularly in ER-positive tumors [53]. In ER+ BC, AR plays a role in inhibiting tumor proliferation. Despite a previous decline in the use of nonsteroidal androgens due to concerns about side effects, recent developments in selective AR modulators (SARMs), such as GTx-024, have renewed interest in AR as a therapeutic target in metastatic BC. For instance, GTx-024, an innovative SARM, shows the potential to slow tumor growth, particularly in ER+ BC [82]. Whole-body AR imaging using 16β-[18F] fluoro-5α-dihydrotestosterone (18F-FDHT) PET/CT emerges as a promising tool for assessing receptor status and managing BC patients. Prior studies have suggested the potential of 18F-FDHT PET as a non-invasive alternative to biopsy for detecting metastases, especially in cases prone to sampling errors [83,84]. These investigations support the use of 18F-FDHT PET as an imaging biomarker for evaluating responses to SARM therapy, such as GTx-024, in ER-positive metastatic BC. However, the limited available data underscore the need for further studies with larger populations to establish definitive conclusions.

##### Others

Several other emerging radiotracers are currently under investigation for BC imaging. These include 3′-deoxy-3′-18F fluorothymidine (FLT) for measuring cell proliferation, (11)C- or (18)F-labeled choline for imaging membrane lipid, and 11C-methionine for evaluating amino acid transport. While these tracers have not yet been integrated into clinical practice, preliminary results suggest their potential to significantly influence clinical approaches. These tracers offer insights into BC biology, aiding in tumor staging, facilitating targeted therapy selection, and monitoring treatment response [85,86]. However, further well-controlled studies are necessary to validate these preliminary findings.

### 1.3. PET/MRI Applications

PET/MRI is an advanced integrated imaging method making its way into clinical practice [87]. FDG PET/MRI combines the metabolic information of FDG PET with the high-resolution soft-tissue characteristics offered by MRI within a single examination. A growing body of evidence from various studies has demonstrated the high value of fused PET/MRI in the diagnosis and staging of breast cancer patients, surpassing both PET/CT and MRI [88,89].

In terms of diagnosis, PET/MRI tends to achieve a higher rate of accurate diagnosis for breast lesions when compared to PET/CT, MRI, and CT [90]. A meta-analysis published in 2019 revealed compelling findings regarding the diagnostic performance of PET/MRI in BC staging: the pooled sensitivity and specificity of 91%, 91% for T-staging, 94%, 90% for N staging, and 98%, 96% for M staging [69].

A very recent meta-analysis conducted by Ruan et al. confirmed the excellent diagnostic performance of PET/MRI in identifying breast lesions and distant metastases in breast cancer, with overall sensitivity and specificity of 93% and 95%, respectively [88]. They reported that while PET/MRI performs very well in diagnosing breast lesions by multiparametric imaging, it may be slightly less effective in nodal assessment but is still much better than MRI and CT. Regarding distant metastasis, PET/MRI demonstrated high accuracy in BC staging, especially for bone lesions where PET/MRI achieved a 100% detection rate. Additionally, with its high soft-tissue resolution and multiple sequences targeting the liver, PET/MRI surpassed PET/CT in detecting liver metastases, especially subcentimetric lesions without FDG uptake. Moreover, PET/MRI proved superior to PET/CT in diagnosing brain metastases. In recurrent BC, a study comparing FDG PET/CT, MRI, PET/MRI, and CT found that whole-body PET/MRI provided the highest diagnostic performance for whole-body staging [91].

Beyond its role in diagnosis, staging, and restaging, PET/MRI may serve as a predictive tool for discerning BC molecular subtypes, predicting therapy response, and determining patient prognosis. For example, it is suggested that high SUVmax is more likely associated with ER/PR negativity. High ADC values and high SUVmax are also more likely linked with HER2 negativity [92]. Furthermore, the SUVmax and ADC values provided by PET/MRI are correlated with the expression of tumor markers, which is of great value for early diagnosis, predicting staging and tumor aggressiveness, and disease prognosis [93]. The integration of PET/MRI data into these aspects of BC assessment adds a layer of precision, contributing to a more comprehensive understanding of the disease and informing tailored therapeutic approaches.

Despite the aforementioned advantages, there are some drawbacks to the application of PET/MRI in BC evaluation. The higher sensitivity of PET/MRI can lead to more false-positive results, comprising inflammatory lesions, papillary adenomas, and fibromas [94]. Additionally, the use of breast-specific coils may hinder a complete assessment of axillary and thoracic nodal disease due to the limited field of view. Furthermore, in terms of M staging, PET/MRI is less sensitive to non-FDG-avid and small pulmonary nodules [95]. Lastly, PET/MRI is expensive, time-consuming, and not routinely applied in clinical practice.

While PET/MRI shows promise as a comprehensive tool for better evaluating breast lesions, lymph nodes or not, and distant metastases compared to PET/CT, further prospective studies are needed before drawing definitive conclusions. Addressing these limitations is crucial to ensuring the reliable and accurate clinical implementation of PET/MRI in BC assessment.

### 1.4. Recent Advancements: Artificial Intelligence, Machine Learning, and Deep Learning

In recent years, there have been significant advancements in artificial intelligence (AI) and machine learning (ML) within the field of oncology, particularly in cancer imaging [96,97]. Numerous studies have highlighted the potential value of AI-based imaging analysis in breast imaging, such as for risk assessment, detection, diagnosis, nodal staging, prognosis, and treatment response [98,99].

Within the realm of screening and diagnosis, deep learning (DL) methods have undergone decades of development to facilitate computer-aided detection of breast lesions across various imaging modalities, such as breast MRI, ultrasound, and mammography [100]. A study conducted by Romeo et al. involving 120 breast lesions (101 malignant and 19 benign) demonstrated the high accuracy of an AI-based radiomics model. This model, incorporating quantitative parameters and radiomics features extracted from simultaneous multiparametric FDG PET/MRI images, outperformed expert readers in discriminating between benign and malignant breast lesions [101]. Similarly, the diagnostic performance of 19 breast radiologists using the AI system to differentiate between malignant and benign lesions on breast MRI was higher than that using a conventional computer-aided detection/diagnosis (CAD) system only, and the sensitivity of BI-RADS grading was also improved from 90% to 94%. Consequently, the integration of AI into functional and metabolic breast imaging holds the potential to support human readers in precisely characterizing suspicious breast lesions, thus obviating the need for unnecessary invasive procedures. Furthermore, DL methods play a crucial role in assessing breast density and parenchymal patterns, offering valuable insights for breast cancer risk assessment and, ultimately, guiding personalized screening strategies [102].

Concerning axillary nodal metastasis, ML prediction models derived from MRI or PET/MRI have demonstrated comparable or superior performance compared to experienced physicians, with the potential advantage of adjusting decision thresholds [97] to achieve low rates of false negatives such that invasive procedures might potentially be omitted.

Apart from computer-based diagnosis and staging, AI applications in breast imaging extend to evaluating molecular subtypes, predicting prognosis, and assessing therapeutic response [14]. These applications provide the capability to generate predictive image-based phenotypes of breast cancer, contributing to the advancement of precision medicine.

Integrating ML models into imaging-based decision-support tools holds significant potential to enhance the diagnostic workup in BC patients, improving patient outcomes while alleviating radiologists’ workloads. However, it is crucial to recognize the existing challenges in this domain, including the need for large and diverse datasets, ensuring model interpretability, and addressing ethical considerations. Despite the approval and evaluation of various DL-based software by regulatory bodies like the FDA and CE marking, this software, while promising, is not yet comprehensive enough to cover the full spectrum of clinical workflows. Several limitations, such as those indicated above, must be overcome before its widespread adoption into clinical practice can be achieved.

## 2. Conclusions

We reviewed the current and potential applications of PET/CT in breast cancer (BC), including the latest advancements in imaging techniques and precision medicine tracers. While FDG remains a foundational radiotracer in BC PET imaging, research into novel biomarkers is yielding promising results. New tracers and theranostic ligands, such as 68Ga-FAPI and 18F-FES, show potential in assessing various tumor behaviors, enhancing detection precision, and hopefully improving therapeutic approaches. Furthermore, advancements in PET technology, including total body PET scanners, PET/MRI imaging, and the integration of AI, ML, and DL methods, offer significant improvements in diagnostic accuracy. These innovations are particularly beneficial in tumor characterization, assessing nodal and occult metastases, and guiding therapeutic decisions. The detailed tumor information provided by these advanced techniques is expected to drive further advancements in BC treatment through more individualized and precise approaches. However, while promising, there remain challenges to their widespread clinical application.

In summary, the future of PET/CT in BC looks promising with the advent of novel biomarkers, advanced imaging technologies, and AI-driven analysis. However, achieving widespread clinical application will require continued research, interdisciplinary collaboration, and rigorous validation. By addressing these challenges, we can move toward more precise, individualized, and effective BC management.

## Abbreviations

Positron emission tomography (PET), 18F-fluorodeoxyglucose (FDG), breast cancer (BC), 16α-18Ffluoro-17β-oestradiol (FES), ultrasound (US), magnetic resonance imaging (MRI), maximum intensity projection (MIP), estrogen receptor (ER), progesterone receptor (PR), human epidermal growth factor receptor 2 (HER2), maximum standardized uptake value (SUVmax), triple-negative breast cancers (TNBCs), National Comprehensive Cancer Network (NCCN), sentinel lymph node biopsy (SLNB), axillary lymph node (ALN), area under the curve (AUC), fine needle aspiration (FNA), neoadjuvant chemotherapy (NAC), pCR (pathological complete response), cancer antigen 15-3 (CA15-3), carcinoembryonic antigen (CEA), fibroblast activation protein (FAP), gastrin-releasing peptide receptor (GRPR), fibroblast activation protein inhibitor (FAPI), cancer-associated fibroblasts (CAFs), 16β-18Ffluoro-5α-dihydrotestosterone (FDHT), androgen receptor (AR), 18Ffluorofuranyl norprogesterone (FFNP), monoclonal antibodies (mAbs), prostate-specific membrane antigen (PSMA), Chemokine Receptor 4 (CXCR4), somatostatin receptors (SSTRs), artificial intelligence (AI), machine learning (ML), deep learning (DL), computer-aided detection (CAD), Food and Drug Administration (FDA).
